# An endoscopic transnasal prelacrimal recess transmaxillary approach to the pterygopalatine fossa and infratemporal fossa

**DOI:** 10.3389/fsurg.2023.1264847

**Published:** 2023-11-16

**Authors:** Jian Liu, Zhijun Yang, Bin Lu, Zhiyong Bi, Pinan Liu

**Affiliations:** ^1^ Department of Neurosurgery, Beijing Tiantan Hospital, Capital Medical University, Beijing, China; ^2^ Department of Neurosurgery, Tangshan Gongren Hospital, Tangshan, China

**Keywords:** prelacrimal recess, pterygopalatine fossa, infratemporal fossa, transmaxillary approach, endoscopic

## Abstract

**Objective:**

In this paper, the goal of the authors is to present the anatomic nuances and their clinical experience with lesions of the pterygopalatine fossa and infratemporal fossa using an endoscopic transnasal prelacrimal recess transmaxillary approach (PLRMA).

**Methods:**

An endoscopic anatomical dissection of three fresh cadaveric heads was performed bilaterally to evaluate the feasibility of the PLRMA. Prior to dissection, stereotactic computed tomography scans were obtained for each head to obtain anatomical measurements. The area of exposure on the posterior wall of the maxillary sinus was determined using stereotaxis. The cases of six patients with schwannomas or epidermoid cysts who underwent the transnasal PLRMA were illustrated.

**Results:**

The mean area of exposure on the posterior wall of the maxillary sinus was 9.55 cm^2^. Total resection was achieved in all six patients. The mean follow-up time was 16 months, and one patient complained of postoperative facial numbness, which resolved gradually. No cases of chronic sinusitis were reported.

**Conclusions:**

The endoscopic transnasal PLRMA provides efficient operative exposure to the pterygopalatine fossa and infratemporal fossa. Preserving the integrity of the mucosa on the nasal lateral wall is an advantage of this approach.

## Introduction

The pterygopalatine fossa (PPF) and infratemporal fossa (ITF) are located behind the maxillary sinus, providing a natural corridor for endoscopy to access these areas (1–6). Various endoscopic approaches to the ITF have been previously described, such as an endoscopic sublabial transmaxillary approach, an endoscopic Denker approach, an endoscopic endonasal transmaxillary approach, and a contralateral transseptal transmaxillary approach (7–9). Although endoscopic approaches are substantially less invasive than conventional approaches, they can still lead to several sequelae, including nasolabial groove collapse, superior alveolar numbness, and over-resection of nasal structures. It is well-known that nasal structures are critical for maintaining the physiological functions of the nasal cavity. An over-resection of nasal structures, such as the middle nasal concha, the inferior nasal concha, the nasal septum, or the total lateral wall of the nose, could severely affect the physiological functions of the nasal cavity and even induce conditions such as empty nose syndrome and depression, imposing heavy psychological and physical burden on patients. The endoscopic transnasal prelacrimal recess transmaxillary approach (PLRMA) is an improved endoscopic endonasal transmaxillary approach that enters the maxillary sinus through a submucosal dissociation of the nasolacrimal duct (10–12). The benefits of this approach include preventing superior alveolar numbness and the development of cosmetic issues, as well as preserving the integrity of mucosa on the nasal lateral wall. However, endoscopic anatomical evidence for the area of exposure and the surgical freedom of this modified approach remains scarce (13, 14). In this study, we aim to evaluate the feasibility of the PLRMA approach to the PPF and ITF and share our surgical experience through case illustrations.

## Materials and methods

The endoscopic transnasal PLRMA was performed bilaterally on three fresh silicon-injected heads. Dissections were carried out using a 30° endoscope, burrs, dissector blades, and standard endoscopic instruments (Kal Storz, Tuttlingen, Germany). Images were captured using an AIDA HD system (Karl Storz, Tuttlingen, Germany). High-resolution computed tomography (CT) scans were performed on each specimen, and the dataset was imported into a surgical navigation system (Ariemedi Medical Technology Co., Ltd, China). A navigation probe was used to identify the boundary of exposure on the posterior wall of the maxillary sinus, and the area of exposure was calculated using a navigation software.

### Endoscopic transnasal prelacrimal recess transmaxillary approach

**Figure 1:**
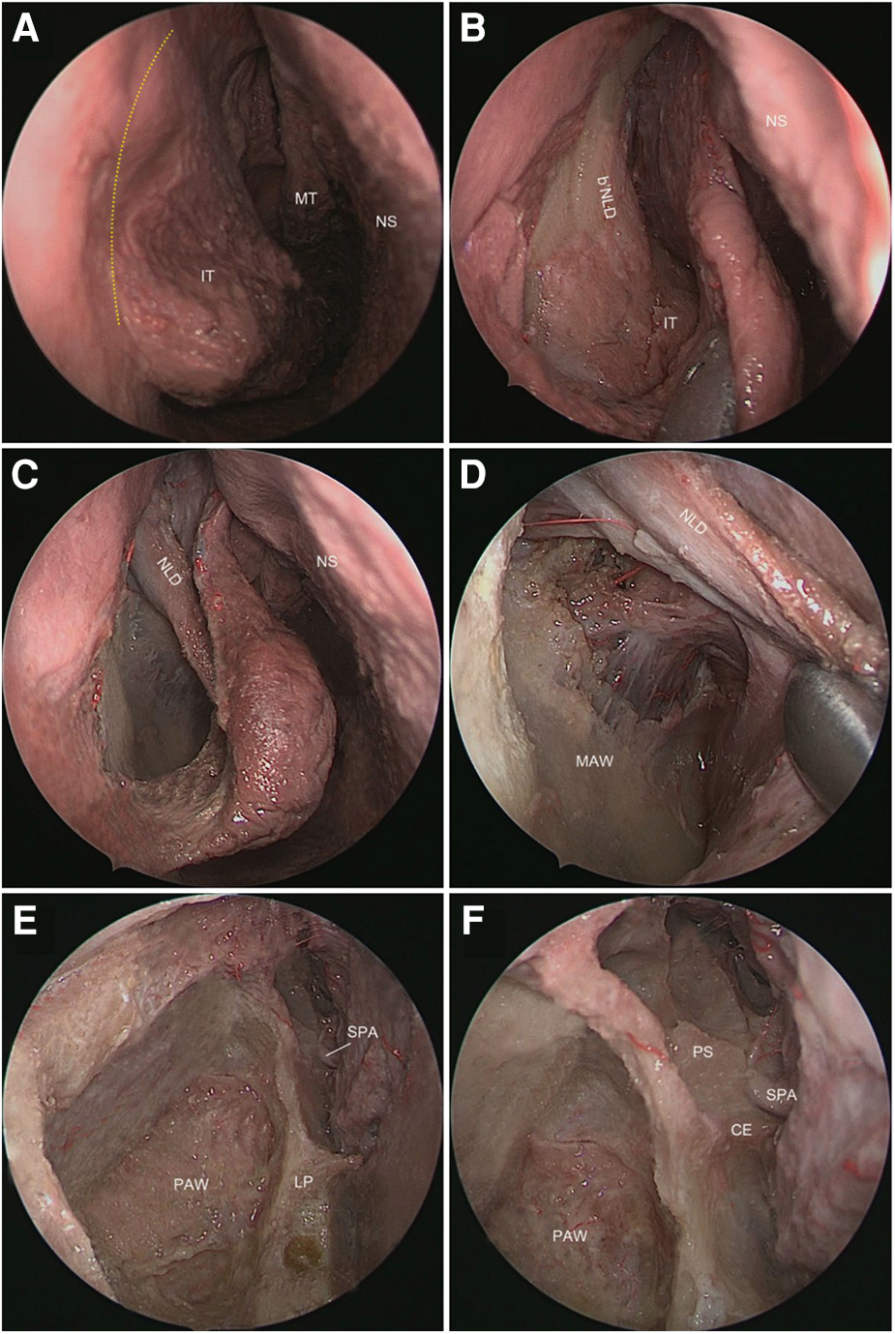
Entry into the maxillary sinus (right nasal cavity). (**A**) The mucosal incision is indicated by the yellow dashed line. (**B**) The mucous membrane of the lateral wall of the nasal cavity was peeled to expose the bNLD and the IT. (**C**) The IT and the bony nasolacrimal duct were drilled out to expose the mucosal nasolacrimal duct (mNLD). (**D**) The mucous flap formed by mNLD and the mucous membrane of the lateral wall of the nasal cavity were elevated to expose the medial antral wall (MAW). (**E**) The MAW and the mucosa in the maxillary sinus were removed to reveal the posterior antral wall (PAW) and lamina perpendicularis (LP). (**F**) A close view of the anterior wall of the pterygopalatine fossa formed by the processus orbitalis and processus sphenoidalis (PS) of the LP. NS, nasal septal; MT, middle turbinate; SPA, sphenopalatine artery; CE, crista ethmoidalis.

An arc incision was made in front of the nasolacrimal duct and the cephalic end of the inferior turbinate (IT). The mucosa was elevated to expose the inferior turbinate and bony nasolacrimal duct (bNLD). The inferior turbinate and bony nasolacrimal duct were carefully removed using the Kal Storz S III neurodrill with a 3-mm diamond burr to reveal the lacrimal sac. The lacrimal sac was freed using Cottle's elevator, and the lacrimal sac-mucous flap was sufficiently elevated in the midline direction. We performed a submucosal medial maxillectomy to gain entry into the maxillary sinus. To standardize the collected data, the grinding range of the medial wall of the maxillary sinus for all the specimens was unified as follows: extending from the frontal process of the maxilla in the front, up to the semilunar hiatus, down to the flat bottom of the nose, and back to the vertical plate of the palatine bone. After entering the maxillary sinus, we peeled away the mucosa and removed the posterior wall of the maxillary sinus, exposing the vertical plate of the palatine bone, the orbital process, and the sphenoid process of the palatine bone (Figure 1). Finally, the posterior wall of the maxillary sinus and the anterior wall of the PPF were removed.

### Area of exposure

**Figure 2:**
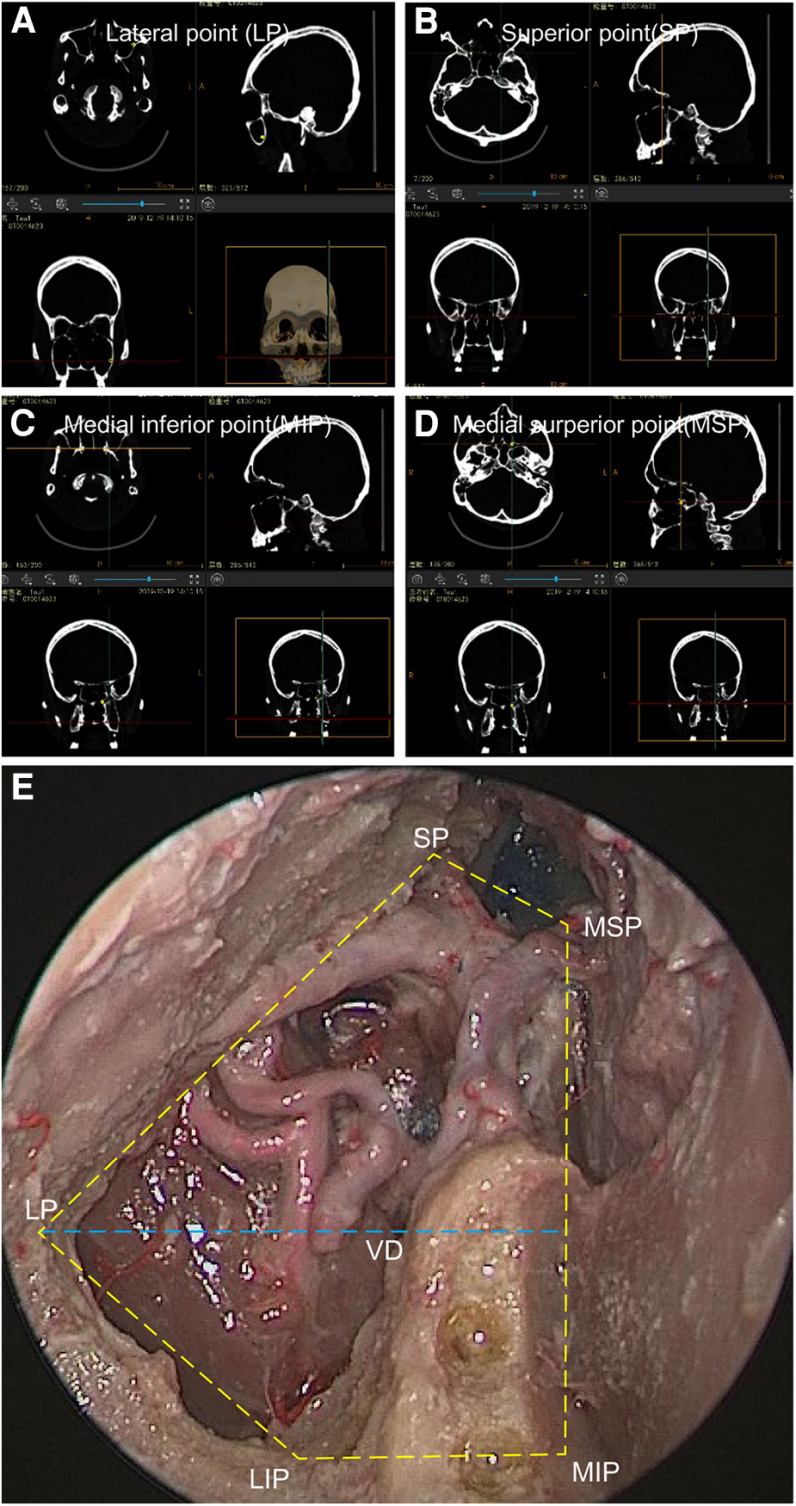
Anatomical landmarks can be used to calculate the area of exposure of the PLRMA. (**A**) The LP on a navigation system. (**B**) The SP on the navigation system. (**C**) The MSP on the navigation system. (**D**) The MIP on the navigation system. (**E**) An endoscopic image from the PLRMA showing the area of the exposure and the landmark points in the right maxillary sinus.

To calculate the area of exposure of the PLRMA, five points on the posterior wall of the maxillary sinus and the anterior wall of the PPF were identified using a navigation probe. Out of these five points, four are fixed anatomical landmarks: (1) The superior point (SP) where the zygomatic nerve enters the infraorbital fissure. (2) The medial superior point (MSP) corresponding to the sphenopalatine foramen. (3) The medial inferior point (MIP) located at the lowest point of the medial surface of the vertical plate of the palatine bone. (4) The lateral inferior point (LIP), which is the most inferior point of the posterior wall of the maxillary sinus. The lateral point (LP) was defined as the most lateral limit point of the operation of the navigation probe on the posterior wall of the maxillary sinus. The pentagonal shape formed by these points delineates the area of exposure of the PLRMA on the posterior wall of the maxillary sinus and PPF. We defined the vertical distance (VD) between the LP and the connection line of the MSP to MIP as the working width. This parameter represents the maximum operating width of the PLRMA from the medial surface of the vertical plate of the palatine bone to the lateral side. Screen captures from a neuronavigation system were utilized to illustrate these five points. A navigation software was then used to calculate the area and width of the pentagon, allowing us to assess the feasibility of the PLRMA (Figure 2).

### Patient population

During the period between January 2020 and April 2022, six patients who underwent the PLRMA for resection of benign tumors in the PPF or ITF were enrolled in this study. Preoperative imaging included a CT scan and/or an MRI study. The follow-up period ranged from 6 to 24 months. Four patients were women and two patients were men. The average age of the patients was 45.8 years, ranging from 24 to 61 years.

## Results

The mean area of exposure of the PLRMA was 9.6 cm^2^, and the mean working width was 3.35 cm (Table 1).

**Table 1:** The mean area of exposure and working width of the PLRMA.

	Specimen 1		Specimen 2		Specimen 3		Means
	Left	Right	Left	Right	Left	Right	
Area of exposure (cm^2^)	9.2	9.4	9.8	9.8	9.5	9.6	9.6
Working width (cm)	3.1	3.2	3.6	3.5	3.3	3.4	3.4

The endoscopic endonasal PLRMA was used in six patients. The average age of patients was 45.8 years (range 24–61 years). The current report included two categories of lesions: schwannoma (four patients) and epidermoid cyst (two patients). All six lesions were confined to the PPF or ITF without extending into adjacent areas. Total resection was achieved in each patient. After the operation, the patients were instructed to wash the nasal cavity with sea salt water 3–5 times a day for 1 month to prevent sinusitis. The mean follow-up time in our series was 16 months. One patient experienced postoperative facial numbness for 3 months. Two patients had mild postoperative sinusitis, characterized by a short-course stuffy nose, which resolved within 2 weeks. None of the six patients developed empty nose syndrome (Table 2).

**Table 2:** Characteristics of six patients in whom an endoscopic endonasal prelacrimal recess transmaxillary approach was used for their lesions.

Case no.	Age, years (sex)	Diagnosis	Site of lesion	Follow-up, months	Complications
1	24 (female)	Schwannoma	ITF	10	None
2	44 (male)	Schwannoma	ITF	18	Mild postoperative sinusitis
3	37 (male)	Schwannoma	PPF	22	Facial numbness
4	61 (female)	Schwannoma	PPF	10	None
5	57 (female)	Epidermoid cyst	ITF	24	Mild postoperative sinusitis
6	52 (female)	Epidermoid cyst	PPF	12	None

### Case illustrates

#### Case 1

**Figure 3:**
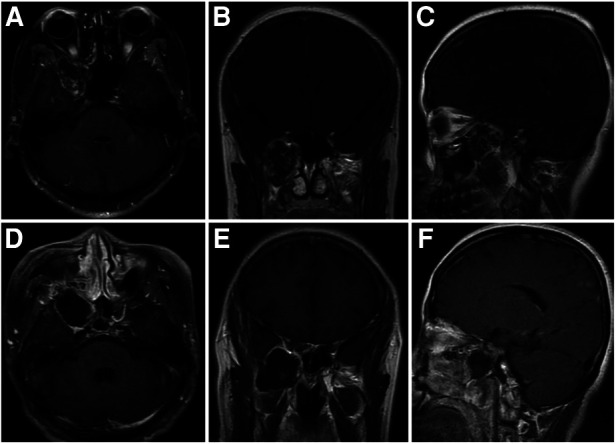
Case 1: a 46-year-old woman with a schwannoma in the PPF. A preoperative T1-weighted enhanced MRI (**A–C**) shows the tumor located in the PPF. A postoperative T1-weighted enhanced MRI confirms the total removal of the tumor by the PRLMA (**D–F**).

**Figure 4:**
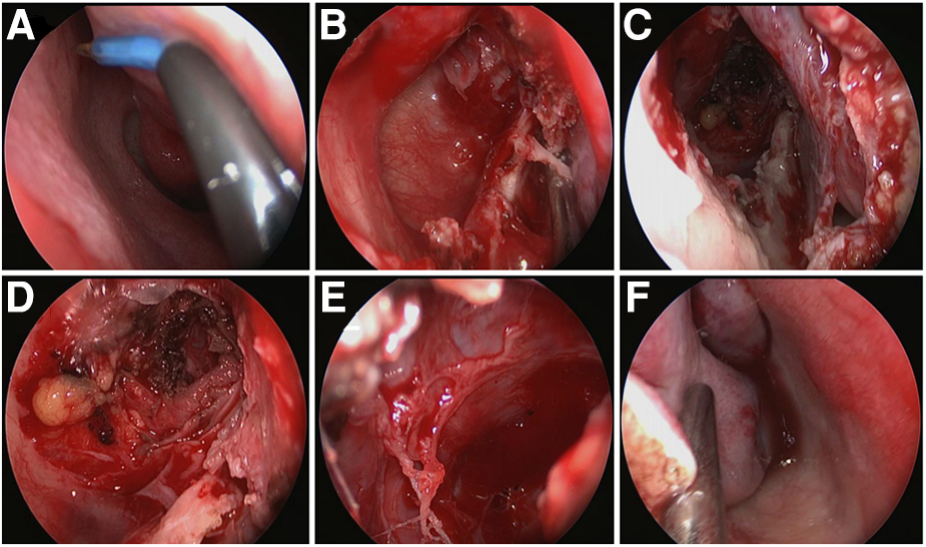
Intraoperative screen capture of Case 1 in the right nasal cavity. (**A**) Incision of the mucosa in front of the nasolacrimal duct eminence. (**B**) Entry into the maxillary sinus through submucosal medial maxillectomy. (**C**) The lacrimal sac-mucous flap is elevated to show the surgical corridor of the PLRMA. (**D**) The screen capture of the maxillary sinus after the tumor shows total resection. (**E**) Close observation of the tumor cavity after complete resection of the tumor. (**F**) The turbinate mucosa is completely preserved after the operation.

A 46-year-old woman presented with right facial numbness. Imaging revealed a 3 cm mass in the PPF (Figure 3). We used an endoscopic transnasal PLRMA. The tumor was visible in the PPF after we gained entry into the maxillary sinus through submucosal maxillectomy. It was fully exposed and removed through this surgical channel, and the turbinate mucosa was completely preserved after the operation (Figure 4). The postoperative pathological diagnosis confirmed the presence of a schwannoma.

#### Case 2

**Figure 5:**
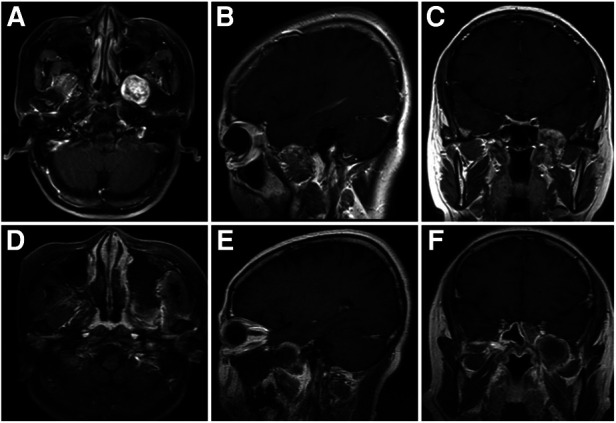
Case 2: a 34-year-old man with a schwannoma in the ITF. A preoperative T1-weighted enhanced MRI (**A–C**) shows the tumor located in the ITF. A postoperative T1-weighted enhanced MRI confirms the total removal of the tumor by the PRLMA (**D–F**). The lateral wall of the nasal cavity remains intact (**D**).

**Figure 6:**
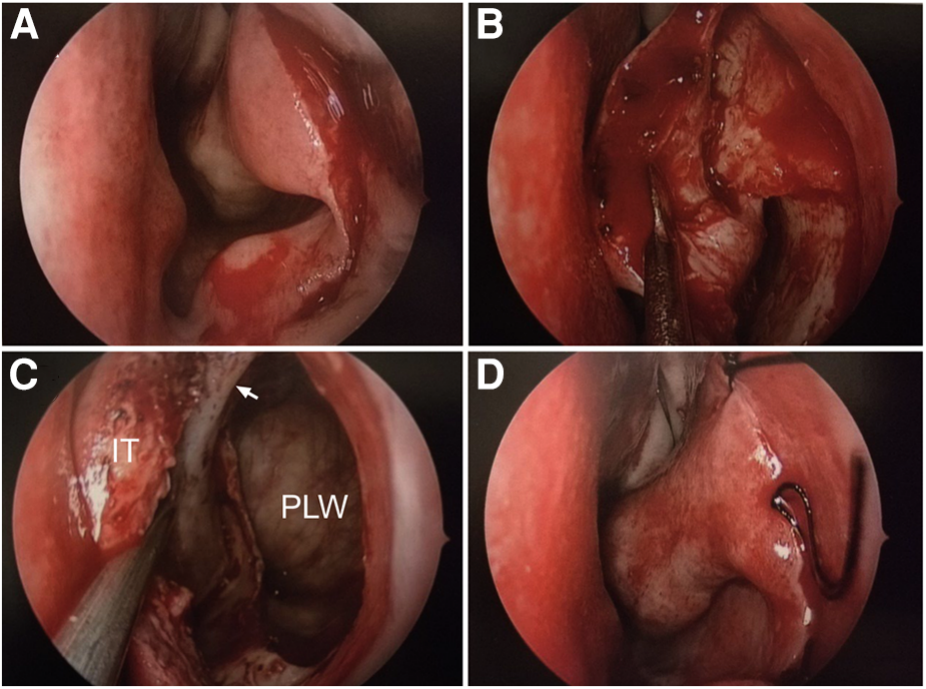
Intraoperative screen capture of Case 2 in the left nasal cavity. (**A**) Incision of the mucosa in front of the nasolacrimal duct eminence. (**B**) The mucosa is elevated to expose the inferior turbinate and bony nasolacrimal duct. (**C**) Entry into the maxillary sinus through submucosal medial maxillectomy. (**D**) The turbinate mucosa is completely preserved after the operation.

A 34-year-old man discovered a lesion in the ITF during a physical examination. The lesion was located behind the posterior wall of the maxillary sinus, next to the lateral plate of the pterygoid process (Figure 5). The PLRMA was performed to access the ITF. The tumor was completely removed and was confirmed to be a schwannoma through pathology. The mucosa of the lateral wall of the nasal cavity remained intact after the operation (Figure 6).

## Discussion

Several variations of endoscopic transmaxillary approaches have been described for accessing the ITF and PPF, such as an endoscopic Caldwell–Luc approach, an endoscopic Denker approach, a contralateral transseptal approach, and a medial maxillectomy approach (14, 15). While the endoscopic transmaxillary approaches can provide adequate exposure in the ITF and PPF, they are often accompanied by various surgical complications, such as superior alveolar numbness, nasolabial groove collapse, and even empty nose syndrome, due to the over-removal of nasal structures. Empty nose syndrome, in particular, can induce severe psychological and physical effects on patients and may even lead to psychological conditions such as anxiety and depression. To reduce the invasiveness of the endoscopic transmaxillary approach, some scholars (16) have proposed modified transmaxillary approaches through the inferior meatus or prelacrimal recess to access the maxillary sinus. Zhou et al. reported the resection of tumors in the PPF and ITF through an endoscopic prelacrimal recess approach (PLRA) (10–12). This modified transmaxillary approach helps gain entry into the maxillary sinus through submucosal medial maxillectomy after freeing the nasolacrimal duct, thereby allowing access to the maxillary sinus, while preserving the integrity of the mucosa of the lateral nasal wall. In addition, this method does not injure the superior alveolar nerves and prevents the development of cosmetic issues. In this study, we evaluated the anatomical rationality of this modified transmaxillary approach, called the endoscopic transnasal prelacrimal recess transmaxillary approach, using endoscopic anatomy and a navigation system.

In this study, five extreme anatomical landmarks were selected on the posterior wall of the maxillary sinus and the anterior wall of the PPF, forming a pentagon. This pentagon represents the area of exposure of the PLRMA. The PLRMA provided seemingly adequate exposure not only of the PPF but also of the most medial aspect of the posterior wall of the maxillary sinus (Figure 2). The differences in the areas of exposure in sublabial anterior maxillectomy and the Denker approach, as well as medial maxillectomy, have been described by Carrau et al. (17, 18) and Little et al. (19, 20), separately. Our measurement of the exposure area was conducted following Little’s method. The mean exposure area of the PLRA was 9.6 cm^2^. Little previously compared the ipsilateral endonasal approach, the Caldwell–Luc approach, and the contralateral endonasal transseptal approach using a navigation probe. The exposure areas of the posterior wall of the maxillary sinus through these approaches were 10.4, 9.9, and 10.0 cm^2^, respectively. A comparison of the data showed that the exposure area of the PLRMA was similar to those of the other approaches. Although we did not conduct a quantitative comparison with other endoscopic transmaxillary approaches, the constraint of the nasolacrimal duct was removed. We believe that the lateral exposure of the PLRMA on the posterior wall of the maxillary sinus will be slightly better than that of the traditional medial maxillectomy.

The mucosa of the lateral nasal cavity serves essential physiological functions such as warming and humidifying the air in the nasal cavity, as well as increasing air resistance. Excessive damage to nasal cavity structures, such as turbinectomy or an extensive resection of the lateral wall of the nasal cavity, can lead to permanent postoperative nasal cavity discomfort in the form of nasal congestion, nasal dryness, pterygopalatine neuralgia, chest distress, wheezing, and even empty nose syndrome. Prolonged discomfort can induce depression in patients. Therefore, greater attention should be given to better preserving the physiological functions of the nasal cavity. While traditional endoscopic transmaxillary approaches offer adequate exposure to the ITF and PPF, they often require sacrificing the turbinates, ethmoidal cells, the posterior part of the nasal septum, and a significant amount of nasal mucosa. The major advantage of the PLRMA lies in preserving the integrity of the nasal cavity mucosa when accessing the maxillary sinus. This is achieved by suturing the mucosa directly at the head end of the inferior concha, preserving the morphology of the turbinate. Therefore, the PLRMA is better suited for protecting the physiological functions of the nasal cavity.

The PLRMA is essentially a modified endoscopic transmaxillary approach that provides access to the maxillary sinus through submucosal medial maxillectomy. Although the advantage of the PLRMA lies in preserving the integrity of the nasal cavity mucosa, as mentioned previously, this approach has limitations concerning its expansion capability on the medial and lateral sides. For tumors of different types and locations, the selection of approaches should be individualized. Giant or invasive malignant tumors such as nasopharyngeal carcinoma and adolescent angiofibroma require an extensive resection of the lateral nasal wall structures, and even multiple approaches need to be used in combination. However, for benign tumors with relatively small sizes, such as the schwannoma and epidermoid cyst, the PLRMA could ensure complete tumor resection, while preserving nasal cavity functions as much as possible. This provides more options for endoscopic access to the ITF and PPF.

## Conclusions

The endoscopic transnasal prelacrimal recess transmaxillary approach can offer sufficient exposure to the pterygopalatine fossa and infratemporal fossa, allowing for the complete preservation of the integrity of the nasal mucosal structure, while avoiding excessive resection of nasal cavity components.

## Data Availability

The raw data supporting the conclusions of this article will be made available by the authors, without undue reservation.
